# Role of civil society in health care: Mechanisms for realizing universal health coverage in vulnerable communities of India

**DOI:** 10.3389/fpubh.2023.1091533

**Published:** 2023-02-22

**Authors:** Anuja Jayaraman, Armida Fernandez

**Affiliations:** Society for Nutrition, Education and Health Action, Mumbai, India

**Keywords:** universal health coverage, civil society organizations, primary health care, community participation, collaboration with health systems, gap-filler

## Abstract

The role of civil society as a partner in the delivery of primary health care is well-established. The pandemic placed a great burden on the existing public health system and civil society stepped forward not only to help the vulnerable population to mitigate challenges that subsequently arose but also to fill the gaps the pandemic exposed in India's health care system. The objective of this paper is to provide mechanisms for realizing universal health coverage by strengthening primary health care from the perspective of civil society. The paper uses examples of efforts of SNEHA, a non-profit organization working on the health of women and children in informal settlements of Mumbai and other civil society organizations working with vulnerable or hard-to-reach populations. We use existing literature, field data, reports and published work over the years. We find that civil society helps the health system to connect with difficult-to-reach populations and achieve wider coverage. They can also build the capacity of frontline staff in the public systems in formal and informal ways. They can recommend ways to change the attitudes and motivations of these workers. Civil society organizations with their close connection with the community can play the part of a “gap-filler” and data messenger. Finally, they can refer people to appropriate health facilities minimizing out-of-pocket expenditure on health.

## Background

In many instances, ranging from financial inclusion to health care, last-mile connectivity is the main challenge. Consider the case of financial inclusion. The Indian central bank, the Reserve Bank of India, actively fostered the business correspondent model with the objective of “increasing banking outreach and ensuring greater financial inclusion” (1). It is a journey that started in January 2006 and it is still a work in progress. In March 2010, there were only 34,174 business correspondents in rural areas and by December 2021 this had increased to 1,844,732. In urban areas, the number of urban locations covered by business correspondents increased from 447 to 1,412,529 during the same period (1). Business correspondents play a crucial role in the progress toward universal financial inclusion by connecting banks and households in geographically under-banked areas. Non-governmental organizations (NGOs) play a similar role in a different context.

Recently, the pandemic placed a great burden on the existing public health system and civil society stepped forward not only to help the vulnerable population to mitigate challenges that subsequently arose but also to fill the gaps the pandemic exposed in India's health care system (2). The number of NGOs working in India has grown significantly since the 1990s. The United Nations recognizes that civil society organizations have a crucial role to play in delivering Sustainable Development Goals (SDGs). The third goal (SDG 3) pertains to ensuring “healthy lives and promote wellbeing for all at all ages.” This is probably among the most ambitious goals in terms of breadth, scale and complexity. Within this, target 3.8 relates to “universal health coverage.” The goal of universal health coverage (UHC) is that everyone should receive the health care they need without facing any financial hardships (3). The Sustainable Development Goal 3 of good health and wellbeing includes achieving UHC which is measured by the progress made in indicator 3.8.1 which monitors the coverage of essential health services and 3.8.2 which captures the out-of-pocket payments made for health services (4). As of 2019 on a scale of 0 to 100, India ranks 61 in the UHC index of service coverage which is indicative of significant challenges remaining in the achievement of the indicator (5).

Additionally, there is an agreement that the primary health care (PHC) approach shapes a viable health care system that is effective, equitable and efficient leading to UHC and the achievement of the Sustainable Development Goals (6). While service coverage has improved in the last 20 years, the proportion of people facing financial hardship due to out-of-pocket health spending has increased. With rising poverty and shrinking incomes resulting from the current global economic recession and health systems struggling to provide continuity of health services, the COVID-19 pandemic is likely to halt the progress made toward UHC, particularly among disadvantaged populations (2). One of the guiding principles to strengthening health systems is active engagement with citizens, communities, civil society and the private sector (7).

Over the years, NGOs have filled the gaps in health care provision by focussing on marginalized and vulnerable populations (8–10). NGOs' partnership with the government system is well established wherein the government relies on NGOs to provide universal coverage in health and education (11). A systematic review of the role of NGOs in progressing toward UHC concluded that NGOs could play an important role along with the government and other key stakeholders (9). This review uses the UHC cube dimension to explore the role of NGOs to identify themes and sub-themes wherein service coverage includes human resources, equipment and public health; population coverage includes community participation and inclusion of poor and vulnerable groups; and financial coverage encompasses insurance development, free charge and provision of loans. NGOs can assist in the provision of PHC through the implementation of programs by providing information on health care schemes, ensuring the participation of community members including disadvantaged and vulnerable populations, training health care workers, using appropriate health technologies and applying a gender lens to health promotion while taking local context into account (8).

Given this context, the objective of this paper is to provide mechanisms for realizing UHC by strengthening primary health care from the perspective of civil society. The paper uses examples of efforts of SNEHA, a non-profit organization working on the health of women and children in informal settlements of Mumbai and other civil society organizations working with vulnerable or hard-to-reach populations. We use existing literature, field data, reports and published work over the years to draw learnings for UHC by studying how civil society organizations integrate into the existing public health infrastructure and at the same time engage with the community.

## Realizing universal health coverage in vulnerable communities

The Primary Health Care Performance Initiative framework outlines the 5S-5M-5C schematic which describes a systematic approach to using appropriate inputs (5S) along with delivery mechanisms (5M) with the aim to achieve set health outcomes (5C) by strengthening primary health care for all (12). This paper uses this framework to explore what functions community-based organizations are capable of performing even when government-run urban health systems are functioning well.

### Inputs (5S) needed for a strong primary health care

In the 5S-5M-5C framework, 5S refers to the inputs including the availability of health care providers, staff, adequate finances, sufficient medical and other supplies and surveillance in terms of responding to community needs (12). During the pandemic, SNEHA got involved in new activities which included the distribution of food items in the community, dissemination of information related to COVID-19 and coordination with public systems for the continuity of routine services (13). As an organization that worked on issues related to the health and nutrition of women and children, SNEHA ended up distributing fresh fruits and vegetables in informal settlements of Mumbai during the pandemic between April and June 2020 when communities were isolated due to strictly-imposed lockdown, an activity that the organization had never undertaken. In the three-month period, 30,000 boxes of fresh fruits and vegetables were distributed to Dharavi residents (14). This was possible because of SNEHA's collaboration with another Mumbai-based philanthropic organization, other NGOs, the Municipal Corporation of Greater Mumbai (MCGM) and local community members and volunteers. SNEHA could motivate community volunteers and private shopkeepers to arrange for food kits for needy and vulnerable populations including pregnant women and daily wage workers. The philanthropic organization coordinated the procurement of food supplies and SNEHA worked closely with MCGM to identify individuals residing in the containment areas. Having worked in Dharavi for over 20 years, SNEHA had the connections and grip on the pulse of the community to understand their immediate needs at the start of the pandemic which brought an unanticipated convergence of stakeholders (14).

Apart from surveillance and knowing community needs, organizations have supported the public health system in strengthening primary health care by the provision of medical staff and training. As a not-for-profit and humanitarian organization, Doctors For You (DFY) focuses on providing medical care and services to vulnerable communities including disaster-hit zones (15). Their key focus area is disaster response and recovery, public health and nutrition and community development initiatives (16). Through their urban slum health centers, DFY provides basic primary and preventive health services which include immunization, antenatal care and dental care. They also train and build capacity for emergency preparedness and response (17). They bring in the key resources needed for managing the health needs of the community.

Some organizations use technology for last-mile connectivity. The mission of the Digital Empowerment Foundation is to “empower marginalized communities in information dark regions to access, consume and produce information online using digital interventions and ICT tools” (18). This is done by providing access to the internet, using digital tools to raise awareness about government entitlements and opportunities including health, accessing critical information and creating a network of digital practitioners to scale up and collaborate (19).

In 2016, SNEHA implemented “the community volunteer strategy” in vulnerable communities of Mumbai within which they recruited and trained unpaid volunteers to help in implementing child health and nutrition programs. The idea was to initially motivate, handhold and support the volunteers with the expectation that over time they would be able to work independently in the communities in absence of SNEHA and also support the health systems, particularly in accessing PHC. An in-depth study of this strategy highlighted that volunteers were able to participate in events and training organized for them, mobilize community members, informally disseminate health information and help community members access health services (20). Over the years, SNEHA has tried to foster capacities within communities to actively participate in interventions that could improve health outcomes. There is also a high level of trust that exists between SNEHA staff and the community. A study examining community perceptions of COVID-19 from informal settlements in Mumbai found that over 90% of community members trusted information they received from health systems and NGO staff during the pandemic (21). As part of a cluster-randomized control trial, paid women health facilitators were engaged by SNEHA to trigger health behavior change related to maternal and newborn health wherein their role was to create strong community groups and encourage discussion on health issues (22). The learning was that local recruitment helped in developing friendships, establishing relationships and negotiating with community members which aided in contextualizing program strategies and eased implementation. A mechanism for ensuring the sustainability of health projects in vulnerable communities is to develop a cadre of unpaid volunteers in the community to carry out program activities and respond to community needs. Thereby filling the gap to attain UHC.

### Mechanism (5M) for delivering primary health care

5M in the 5S-5M-5C schematic comprises a multidisciplinary team, motivation to work, use of information systems to track indicators, application of data to make evidence-based decisions, and putting in place a well-functioning facility and population health management systems (12). Working on the premise that timely obstetrics care is crucial in preventing both maternal and child deaths, SNEHA has partnered with public health systems of the Mumbai Metropolitan Region to initiate a formal maternity referral network among health facilities. SNEHA's role among other things is to establish referral linkages between sending and receiving facilities, manage referral data using the referral slips, customize clinical protocols to decide types of cases that can be managed by the health facility, train staff and set up referral meetings among health facilities. Patient referral is not new to the medical field however, the need arose when a situational analysis conducted by SNEHA indicated gaps in the referral system. This project was piloted in two regions of the Municipal Corporation of Greater Mumbai in 2004 and over the years scaled up to seven other regions of MMR. Over the years, SNEHA has built the capacity of the health facility to run this referral system independently. The process of complete handover is on-going. Appropriate maternal referral widens the range of services provided to a pregnant woman and adds to the depth of UHC. The aim here was to offer quality delivery services within the government health care system.

In India, an organization like Haqdarshak seeks to leverage technology with to ensure “last-mile” service delivery of government welfare and financial services including health insurance schemes (23). The idea is to bridge the information gap and enable the common man to access health services and difficult-to-comprehend government schemes *via* agent-based service delivery (training field cadres who provide services for a fee) or direct-to-beneficiary services (24).

As an innovative way to address the problem of malnutrition among children, SNEHA implemented the Community-based Management of Acute Malnutrition program between 2011 and 2016 which was part of a large-scale program conducted in partnership with national (Integrated Child Development Services) and municipal government (Municipal Corporation of Greater Mumbai) wherein wasting among children under age 3 decreased by 28% in intervention areas and by only 5% in comparison areas between November 2014 and December 2015 (25). To the best of our knowledge, this was the only large-scale community-based nutrition program to identify, treat, and prevent wasting in urban informal settlements of India and this was possible as at each step efforts by the non-profit organization were taken to strengthen and work in collaboration with the public systems (26).

### Provision of high-quality primary health care services (5C)

5C includes outcomes such as access to health care services, long-term patient-provider relationships, effective transfer of information within the system, ensuring proper promotive, preventive and curative services for all and offering person-centered care (12). During the second wave of the pandemic in India, SNEHA was involved in the provision of COVID-19 vaccines which were offered to everyone above 18 years of age. However, these vaccines were not immediately available in public hospitals and those living in slum communities in most cases could not afford to pay nor could they easily sign-up on the Government's CoWIN application which is the web portal for COVID-19 vaccination registration. In addition, there was vaccine hesitancy which prompted SNEHA to create awareness about the vaccine among community members and also organize vaccination drives to make them available through Public-Private Partnerships. SNEHA's role was to disseminate information and mobilize community members to access vaccines, the private sector procured and administered the vaccines and the government provided the guidelines (27). This initiative resulted in close to 30,000 community members residing in SNEHA's intervention areas receiving COVID-19 vaccines between August and November 2021.

At times appropriate promotive, preventive and curative health services need to be taken to the community's doorstep. Society for Education, Action and Research in Community Health (SEARCH), a non-profit organization established in one of the most impoverished districts in India, works with the tribal community to find solutions to health problems by preventing and treating diseases, imparting knowledge, research and advocacy (SEARCH 2020). Their vision is to work together with the community, live with them, identify the health issues they are facing and find solutions together so that community takes ownership of their own health (28). Their people-centered approach helped them work effectively in an area where government health services were not easily available.

Similar to SEARCH, during the lockdown SNEHA volunteers served as an extension arm for SNEHA's work in the community and helped the community (13). They put additional time into volunteering to disseminate COVID-19-related information in the community, counseling community members to get tested and vaccinated, access public health services during the pandemic, promoting basic hygiene practices in the community, distributing food and conducting door-to-door surveys to identify community members that need help (29). Unpaid community volunteers supported SNEHA's community health workers in carrying-out program activities in containment zones when entry into the implementation area was restricted. Over the years SNEHA has built strong partnerships with public systems such as the Integrated Child Development Services (ICDS) and Municipal Corporation of Greater Mumbai. However, there are real-world challenges in operationalizing cross-sector convergence which could have implications for the provision of primary health care (30). An in-depth study on perspectives and experiences of this collaboration indicated that effort goes into establishing and sustaining personal relationships with staff working in the public system, building trust, training their staff from time to time, disseminating information on public sector schemes, support in conducting routine activities including community mobilization, referrals, data collection and sharing which helps in improving service provision and connecting the services to the community members (31). Thus, enabling access to public health services and social protection schemes which impact out-of-pocket expenditure could reduce the financial burden on individuals.

## Discussion

Over the last 40 years, there has been sizable improvement in maternal, neonatal and child health but this progress has been uneven across and within nations (5). A global shift is also seen in disease patterns and demographics. Currently, over 70% of deaths worldwide can be attributed to non-communicable diseases including hypertension, cancer, diabetes and chronic respiratory diseases (32). The three dimensions of UHC include service, finance and population coverage which keep altering not only with changes in demographic, epidemiological and technological trends but are also affected by the expectations of the people (33). In addition in India, the private sector accounts for over 70% of the total health care expenditure and 76% of the population does not have health insurance (34) which means a high out-of-pocket expenditure for the patients.

The Global Conference on primary health care in Astana, Kazakhstan in October 2018 endorsed that “strengthening primary health care (PHC) is the most inclusive, effective and efficient approach to enhance people's physical and mental health, as well as social wellbeing, and that PHC is a cornerstone of a sustainable health system UHC and health-related Sustainable Development Goals” which emphasizes the notion of intersectoral approach (35).

The Global Action Plan for Healthy Lives and Wellbeing for All (SDG3 GAP) seeks to find “new ways of working, building on existing successful collaborations.” And civil society is central to these collaborations.

Two important key takeaways are the following. One, local civil society organizations are trusted resources in communities and are best positioned to identify community needs. Second, they could play an important role in leveraging existing networks and integrating services to serve the health needs of vulnerable populations and looking for innovative tech-based solutions to address future health challenges.

Beyond undertaking interventions, civil society can perform a range of functions to make the progress toward universal health coverage tractable: analyse budgetary allocations, track relevant indicators at the national and local levels and undertake research at the local level to help the governments in planning, etc. In the provision of PHC services, civil society organizations could either work jointly with the public systems or complement their work. They could be the “gap-filler” where they facilitate the delivery of public health services and ensure better outreach and community engagement (31). Thus, making cross-sector convergence possible. In India, civil society could seek to ensure the “last-mile” service delivery of government welfare and financial schemes. For example, SNEHA projects have good community outreach and a network of volunteers which has enabled it to continue its work during the pandemic and contribute to the COVID relief work in collaboration with systems and other partners.

We find that civil society helps the health system to connect with difficult-to-reach populations and achieve wider coverage. They can also build the capacity of frontline staff in the public systems in both formal and informal ways. They can recommend ways to change the attitudes and motivations of these workers. Civil society organizations with their close connection with the community can play the part of a “gap-filler” and data messenger. They can ensure community mobilization and engagement. Finally, they can refer community members to appropriate health facilities minimizing out-of-pocket expenditure on health. Attaining UHC by 2030 seems like a distant dream but the “third sector” could accelerate the rate at which “people have access to the health services they need, when and where they need them, without financial hardship” in India.

## Data availability statement

The original contributions presented in the study are included in the article/supplementary files, further inquiries can be directed to the corresponding author.

## Author contributions

AJ and AF have contributed to the conception, design of the paper, and to the writing and review of the manuscript. Both authors contributed to the article and approved the submitted version.

## References

[1] Reserve Bank of India. Reserve Bank of India Annual Report. Mumbai: Reserve Bank of India (2022).

[2] United Nations. The Sustainable Development Goals Report. (2021). Availabl online at https://unstats.un.org/sdgs/report/2021/The-Sustainable-Development-Goals-Report-2021.pdf (accessed November 6, 2022).

[3] World Health Organization. Tracking Universal Health Coverage: 2021 Global Monitoring Report. (2022). Available online at: https://www.who.int/publications-detail-redirect/9789240040618 (accessed November 5, 2022).

[4] United Nations. Global Indicator Framework for the Sustainable Development Goals Targets of the 2030 Agenda for Sustainable Development. (2022). Available online at: https://unstats.un.org/sdgs/indicators/Global%20Indicator%20Framework%20after%202022%20refinement_Eng.pdf (accessed November 6, 2022).

[5] Sachs J Lafortune G Kroll C Fuller G Woelm F. Sustainable Development Report 2022, 1st ed. Cambridge: Cambridge University Press (2022). Avilable online at: https://www.cambridge.org/core/product/identifier/9781009210058/type/book (accessed November 6, 2022).

[6] World Health Organization. Universal Health Coverage: Primary Health Care towards Universal Health Coverage - Report by the Director-General. SEVENTY-SECOND WORLD HEALTH ASSEMBLY (A72/12), Provisional agenda item 11.5. (2019). Available online at: https://apps.who.int/gb/ebwha/pdf_files/WHA72/A72_12-en.pdf (accessed November 6, 2022).

[7] World Health Organization. Healthy Systems for Universal Health Coverage: A Joint Vision for Healthy Lives. Washington, DC: World Bank (2018). Available online at: https://openknowledge.worldbank.org/handle/10986/29231 (accessed November 5, 2022).

[8] Anbazhagan S Surekha A. Role of non-governmental organizations in global health. Int J Community Med Public Health. (2017) 3:17–22. 10.18203/2394-6040.ijcmph2015154421051475

[9] Sanadgol A Doshmangir L Majdzadeh R Gordeev VS. Engagement of non-governmental organisations in moving towards universal health coverage: a scoping review. Global Health. (2021) 17:129. 10.1186/s12992-021-00778-1 34784948 PMC8594189

[10] Thara R Patel V. Role of non-governmental organizations in mental health in India. Indian J Psychiatry. (2010) 52(Suppl 1):S389–95. 10.4103/0019-5545.69276 21836712 PMC3146177

[11] Karkee R Comfort J. NGOs, foreign aid, and development in Nepal. Front Public Health. (2016) 4:177. 10.3389/fpubh.2016.00177 27606310 PMC4995364

[12] Bitton A Veillard JH Basu L Ratcliffe HL Schwarz D Hirschhorn LR. The 5S-5M-5C schematic: transforming primary care inputs to outcomes in low-income and middle-income countries. BMJ Global Health. (2018) 3(Suppl 3):e001020. 10.1136/bmjgh-2018-001020 30305941 PMC6169658

[13] Ramani S Bahuguna M Shaikh N Sridhar R Jayaraman A. A Qualitative Examination Of The Mission Dharavi Project. (2021). Available online at: https://snehamumbai.org/wp-content/uploads/2021/11/mission-dharavi-ualitative-assessment-final.pdf (accessed November 6, 2022).

[14] Chakraborty P. Distribution of Fresh Fruits Vegetables in Dharavi, India: Lessons Learnings from the COVID−19 Pandemic. (2020). Available online at: https://snehamumbai.org/wp-content/uploads/2020/09/Documentation_of_Fruits_Vegetables_Distribution_Project_Dharavi_Sept_2020.pdf (accessed November 6, 2022).

[15] Doctors, For You. About Doctors for You. Available online at: https://doctorsforyou.org/about-us.php (accessed January 25, 2023).

[16] Brochure. Doctors For You. (2021). Available online at: https://doctorsforyou.org/DFY-Brochure.pdf

[17] Organizational Profile. Doctors For You. (2020). Available online at: https://doctorsforyou.org/files/Organizational-Profile-DFY-2020.pdf

[18] About, Digital Empowerment Foundation. Digital Empowerment Foundation. Available online at: https://www.defindia.org (accessed January 25, 2023).

[19] Annual Reports Digital Empowerment Foundation. Di*gital Empowerment Foundation, DEF*. Available online at: https://www.defindia.org/annual-reports/ (accessed January 25, 2023).

[20] Ramani S Shaikh N Jayaraman A. Institutionalizing Community Participation Processes in Urban Informal Settlements: Lessons Learnt from Community Volunteers in SNEHA's Child Health Nutrition (AAHAR) Program (2016-18). (2019). Available online at: https://snehamumbai.org/wp-content/uploads/2019/08/VolunteersStudyReport_2019.pdf (accessed November 6, 2022).

[21] Ramani S Bahuguna M Tiwari A Shende S Waingankar A Sridhar R. Corona was scary, lockdown was worse: a mixed-methods study of community perceptions on COVID-19 from urban informal settlements of Mumbai. PLoS ONE. (2022) 17:e0268133. 10.1371/journal.pone.0268133 35522676 PMC9075633

[22] Alcock GA More NS Patil S Porel M Vaidya L Osrin D. Community-based health programmes: role perceptions and experiences of female peer facilitators in mumbai's urban slums. Health Educ Res. (2009) 24:957–66. 10.1093/her/cyp038 19651641 PMC2777946

[23] About, Haqdarshak. Haqdarshak. Available online at: https://haqdarshak.com/about_hq/ (accessed January 25, 2023).

[24] Reports Compliances. Haqdarshak. Available online at: https://haqdarshak.com/reports_and_compliances/ (accessed January 25, 2023).

[25] Shah More N Waingankar A Ramani S Chanani S D'Souza V Pantvaidya S. Community-based management of acute malnutrition to reduce wasting in urban informal settlements of Mumbai, India: a mixed-methods evaluation. Glob Health Sci Pract. (2018) 6:103–27. 10.9745/GHSP-D-17-00182 29602868 PMC5878065

[26] Chanani S Waingankar A Shah More N Pantvaidya S Fernandez A Jayaraman A. Effectiveness of NGO-government partnership to prevent and treat child wasting in urban India. Mater Child Nutr. (2019) 15(Suppl 1):e12706. 10.1111/mcn.12706 30748121 PMC7198940

[27] Bahuguna M Jayaraman A. Covid-19 Vaccination Drive in Informal Settlements of Mumbai, India: Documenting Processes Lessons Learned. (2022). Available online at: https://snehamumbai.org/wp-content/uploads/2022/07/COVID-19-Vaccination-Drive-Report-Final-1.pdf (accessed November 6, 2022).

[28] Who We Are | Search For Health. Available online at: https://searchforhealth.ngo/who-we-are/ (accessed January 25, 2023).

[29] Bahuguna M Ramani S Jayaraman A Shaikh N. A Tribute to COVID “Yoddhas”: Stories of Community-Led Action from Urban Informal Settlements in Mumbai. (2022). Available online at: https://snehamumbai.org/wp-content/uploads/2022/08/A-tribute-to-COVID-Yoddhas.pdf (accessed November 6, 2022).

[30] Ramani S Sridhar R Shende S Manjarekar S Patil S Pantvaidya S. Implementing a ‘convergent' framework of action against childhood malnutrition in urban informal settlements of mumbai: frontline perspectives. J Fam Med Prim Care. (2021) 10:3600–5. 10.4103/jfmpc.jfmpc_2526_20 34934653 PMC8653499

[31] Ramani S Sridhar R Jayaraman A. Working across Sectors: Exploring Convergence between SNEHA, ICDS MCGM. (2019). Available online at: https://snehamumbai.org/wp-content/uploads/2023/02/Working-across-sectors_fulldraftreport_Aug-2019.pdf (accessed February 8, 2023).

[32] World Health Organization. Noncommunicable Diseases Progress Monitor 2020. (2020). Available online at: https://www.who.int/publications-detail-redirect/9789240000490 (accessed November 5, 2022).

[33] Ketevan G Mamuka N Triin H. [Can People Afford to Pay for Health Care? New Evidence on Financial Protection in Georgia]. Copenhagen: World Health Organization (2021). Regional Office for Europe. Available online at: https://apps.who.int/iris/handle/10665/342814 (accessed November 6, 2022).

[34] Ministry of Health Family Welfare. National Health Estimates (2018-19) Released. (2022). Available online at: https://pib.gov.in/PressReleasePage.aspx?PRID=1858770#:~:text=NHA%20findings%20also%20indicate%20that,1815%20in%202018%2D19 (accessed November 7, 2022).

[35] World Health Organization. Declaration of Astana: Global Conference on Primary Health Care. (2019). Available online at: https://apps.who.int/iris/bitstream/handle/10665/328123/WHO-HIS-SDS-2018.61-eng.pdf?sequence=1andisAllowed=y (accessed November 6, 2022).

